# Supplementary material for: Tocopherol more bioavailable than tocopheryl-acetate as a source of vitamin E for broilers
Source: PLoS One. 2022 May 25;17(5):e0268894. doi: 10.1371/journal.pone.0268894 (PMC9132266; doi:10.1371/journal.pone.0268894)
Supplement: Attachment — Submitted filename: comments editor.docx [file pone.0268894.s002.docx]

2. We note that the grant information you provided in the ‘Funding Information’ and ‘Financial Disclosure’ sections do not match.

When you resubmit, please ensure that you provide the correct grant numbers for the awards you received for your study in the ‘Funding Information’ section.

**** this trial was funded internally by the company three of the authors work for. The system did not allow me to enter that. Hence, I entered a dummy number where a grant number was required.**

We note that one or more of the authors is affiliated with the funding organization, indicating the funder may have had some role in the design, data collection, analysis or preparation of your manuscript for publication; in other words, the funder played an indirect role through the participation of the co-authors. If the funding organization did not play a role in the study design, data collection and analysis, decision to publish, or preparation of the manuscript and only provided financial support in the form of authors' salaries and/or research materials, please do the following:

a) Review your statements relating to the author contributions, and ensure you have specifically and accurately indicated the role(s) that these authors had in your study. These amendments should be made in the online form.

b) Confirm in your cover letter that you agree with the following statement, and we will change the online submission form on your behalf: 
“The funder provided support in the form of salaries for authors [insert relevant initials], but did not have any additional role in the study design, data collection and analysis, decision to publish, or preparation of the manuscript. The specific roles of these authors are articulated in the ‘author contributions’ section.

**** not sure what to say here; 3 of the authors are employed by the funding company, and the research was carried out in house (but supported by sample analysis by UGent). The trial itself was done on request of the employer with the aim as outlined in the paper, and the analyses were carried out to test that aim. The trial was designed and analyzed by TvK; JH wrote the protocol, SBP was responsible for the animal trial. SDS was responsible for some of the vit. E analyses and provided input on trial design and interpretation.**

4. Thank you for stating the following in the Competing Interests section: 
[van Kempen, Punal, and Huijser are employed by the company that funded the work].

Please confirm that this does not alter your adherence to all PLOS ONE policies on sharing data and materials, by including the following statement: "This does not alter our adherence to  PLOS ONE policies on sharing data and materials.” (as detailed online in our guide for authors http://journals.plos.org/plosone/s/competing-interests).  If there are restrictions on sharing of data and/or materials, please state these. Please note that we cannot proceed with consideration of your article until this information has been declared.

Please include your updated Competing Interests statement in your cover letter; we will change the online submission form on your behalf.

**** data can be shared**

5. We note that you have stated that you will provide repository information for your data at acceptance. Should your manuscript be accepted for publication, we will hold it until you provide the relevant accession numbers or DOIs necessary to access your data. If you wish to make changes to your Data Availability statement, please describe these changes in your cover letter and we will update your Data Availability statement to reflect the information you provide.

**** data can be shared**

Additional Editor Comments:

Authors should explain why such period of the study was chosen;

- **Most vitamin E dose response studies measure responses over a much longer period of time. A confounding factor in such experiments is that tissues can become saturated. We wanted to see if by using a very short feeding period we could get good quality data without suffering from such saturation. The actual feeding period needed for this, though, wasn’t clear. Hence, we tried both 2 and 4 days. Text expanded on this**

next - the composition of premixes should be given;

- **The premix was the same for each period; this is corrected. In the originally submitted manuscript we had included micromineral levels in the final feed rather than in the premix; due to differences in feedstuff composition this resulted in small differences in final levels for some of the minerals (e.g., Cu was 21 ppm in the starter, and 20 ppm in the test feed; for other micro minerals the difference is even smaller).**
- **Note: we did have a typo for the vitamin D level; this is corrected.**

how the studied doses were chosen, based on what;

- **80 ppm is at the high end of what is commercially used. Hence, we used that as an upper limit for both treatment groups (clarified in the text). Next, we hypothesized that tocopherol may well be more effective at raising tissue levels, so we allowed for a larger step (factor 2) downward than for tocopheryl-acetate (step 1.5). For tocopheryl-acetate this results in a lower inclusion limit of 16 ppm, which is just above the NRC recommendation for broilers and at the lower end used commercially. For tocopherol, we ended up below the NRC recommendation. Text is expanded on this**.

the production performance should be given.

- **A table was added as requested. Given the very short test period, we wonder if it is indeed valid to present these numbers, but we adhere to the request from the editor.**

**Comments to the Author**

1. Is the manuscript technically sound, and do the data support the conclusions?

The manuscript must describe a technically sound piece of scientific research with data that supports the conclusions. Experiments must have been conducted rigorously, with appropriate controls, replication, and sample sizes. The conclusions must be drawn appropriately based on the data presented.

Reviewer #1: Yes

2. Has the statistical analysis been performed appropriately and rigorously?

Reviewer #1: I Don't Know

- **Sorry for the confusion 😉. We had hoped that by presenting various options that the conclusion remained clear, but also, that the data may well reveal more info than is apparent at first sight.**

3. Have the authors made all data underlying the findings in their manuscript fully available?

The [PLOS Data policy](http://www.plosone.org/static/policies.action#sharing) requires authors to make all data underlying the findings described in their manuscript fully available without restriction, with rare exception (please refer to the Data Availability Statement in the manuscript PDF file). The data should be provided as part of the manuscript or its supporting information, or deposited to a public repository. For example, in addition to summary statistics, the data points behind means, medians and variance measures should be available. If there are restrictions on publicly sharing data—e.g. participant privacy or use of data from a third party—those must be specified.

Reviewer #1: Yes

4. Is the manuscript presented in an intelligible fashion and written in standard English?

PLOS ONE does not copyedit accepted manuscripts, so the language in submitted articles must be clear, correct, and unambiguous. Any typographical or grammatical errors should be corrected at revision, so please note any specific errors here.

Reviewer #1: Yes

5. Review Comments to the Author

Please use the space provided to explain your answers to the questions above. You may also include additional comments for the author, including concerns about dual publication, research ethics, or publication ethics. (Please upload your review as an attachment if it exceeds 20,000 characters)

Reviewer #1: Comments to the Authors of manuscript number: PONE-D-22-00873 entitled „Tocopherol more bioavailable than tocopheryl-acetate as a source of vitamin E for broilers”.
Major concerns:
Line 24-25: In addition, two test procedures were evaluated in which animals received the test substances for either 2 or 4 days only. Why was this done? Please explain.

- **Most trials use a long test period during which tissues can become saturated (and on top, a lot of sample material is needed); we wanted to see if using a short test we could get valuable data. As we had no info on what period to test for, we set up 2 trials in parallel with different conditions (2 and 4 days). This has been clarified in the text.**
- **Note that we also considered a pharmacokinetics trial (single oral dose and tracking tissue responses). This would, however require cannulating the birds which practically is tricky. The approach evaluated yielded ‘cool’ data, and deserves a serious look for future studies. TvK already did a vitamin D study with the same model which also gave good quality data.**

Linia 99-100: what about the content of vitamins in the starter and grower premixes? Was it the same?

**** yes, we used the same premix for each trial phase; this is clarified**

Line 112: “…supplied at 5 different levels…” – what levels exactly? The experimental setup should be presented in a table, as it is illegible in the present form. The choice and level of the factor should be explicit and clear. What was the basis for the choice of these levels?

**** clarified in the text why we used the different doses, and the exact doses are included as well**.

Line 117-118: “…which the test components (contained in a premix)....” - In the composition of the premixes, the amounts or levels of the tested additives should be specified below the table or separately in another table.

**** we had 2 premixes; one as routine in feed manufacturing with a range of vitamins and microminerals (composition specified, but excluding vitamin E), and a 2^nd^ one containing the test product which consisted of the basal diet and the test components. To avoid confusion, the 2^nd^ one is not any more referred to as premix in the text.**

Line 172: “Serum vitamin E levels were well in line with expectations.” Why was it done if the result was known in advance?

**** As explained in the text, the absolute values we obtained fit with what others see in dose response studies. This is very much intended as a validation of the procedures used, not as a conclusion with respect to relative bioavailability.**

Line 284-285: “No efforts were made in this study to assess the impact of vitamin E on for example
oxidative stress. As such, no recommendations can be made on optimal inclusion.” It is a pity. There are also no production performance results indicated by the research, which would increase the value of the study.

**** This is a choice we made; the objective was bioavailability comparison. As the form absorbed is not different, arguably bio efficacy would ‘simply’ be driven by tissue concentration, not by form. It should be noted that performance does not respond well to vitamin E, so performance is a very difficult parameter for assessing vit. E bioefficacy.**

In the discussion, the authors focus on comparison of their research results with studies conducted by other authors. There is no precise interpretation of the results, which is necessary to regard the publication as valuable. Even when the authors try to explain the results of their research, they actually repeat the information contained in the literature they refer to. There is no broader view of the problem. Therefore, I believe that this section is insufficient and needs improvement.

**** We have difficulty placing this comment. The data obtained were analyzed three different ways and each of these analyses was interpreted. Findings from these analyses are indeed compared to what others found and we have highlighted both the overlap and the differences. The findings that T-Ac appears ineffective at low doses to raise tissue levels, and appears again ineffective to raise tissue levels when dosed above 50 ppm are certainly novel. Also, there are few studies that compare bioavailability of T-Ac to T. Hence, we feel this study is certainly novel.**
